# M6A modification regulates tumor suppressor DIRAS1 expression in cervical cancer cells

**DOI:** 10.1080/15384047.2024.2306674

**Published:** 2024-02-19

**Authors:** Yu-Yan Wang, Lian-Hua Ye, An-Qi Zhao, Wei-Ran Gao, Ning Dai, Yu Yin, Xin Zhang

**Affiliations:** a Department of Obstetrics and Gynecology, The First Affiliated Hospital of Jinzhou Medical University, Jinzhou, Liaoning, China; b Department of Internal Medicine, Zigong Fourth People’s Hospital, Zigong, Sichuan, China; c Department of Obstetrics and Gynecology, Xuanwu Hospital, Capital Medical University, Beijing, China; d Department of Oncology, The First Affiliated Hospital of Jinzhou Medical University, Jinzhou, Liaoning, China; e Operating Rooms, The First Affiliated Hospital of Jinzhou Medical University, Jinzhou, Liaoning, China

**Keywords:** Cervical cancer, DIRAS1, epigenetic regulation, m6A

## Abstract

DIRAS family GTPase 1 (DIRAS1) has been reported as a potential tumor suppressor in other human cancer. However, its expression pattern and role in cervical cancer remain unknown. Knockdown of DIRAS1 significantly promoted the proliferation, growth, migration, and invasion of C33A and SiHa cells cultured *in vitro*. Overexpression of DIRAS1 significantly inhibited the viability and motility of C33A and SiHa cells. Compared with normal cervical tissues, DIRAS1 mRNA levels were significantly lower in cervical cancer tissues. DIRAS1 protein expression was also significantly reduced in cervical cancer tissues compared with para-cancerous tissues. In addition, DIRAS1 expression level in tumor tissues was significantly negatively correlated with the pathological grades of cervical cancer patients. DNA methylation inhibitor (5-Azacytidine) and histone deacetylation inhibitor (SAHA) resulted in a significant increase in DIRAS1 mRNA levels in C33A and SiHa cells, but did not affect DIRAS1 protein levels. FTO inhibitor (FB23–2) significantly down-regulated intracellular DIRAS1 mRNA levels, but significantly up-regulated DIRAS1 protein levels. Moreover, the down-regulation of METTL3 and METTL14 expression significantly inhibited DIRAS1 protein expression, whereas the down-regulation of FTO and ALKBH5 expression significantly increased DIRAS1 protein expression. In conclusion, DIRAS1 exerts a significant anti-oncogenic function and its expression is significantly downregulated in cervical cancer cells. The m6A modification may be a key mechanism to regulate DIRAS1 mRNA stability and protein translation efficiency in cervical cancer.

## Introduction

Cervical cancer is considered almost entirely preventable because of highly effective primary (HPV vaccine) and secondary (screening) prevention measures.^1^ In 2018, the Director-General of the World Health Organization (WHO) called for global action to eliminate cervical cancer (≤4 per 100,000 women globally) through a triple-intervention strategy, that is projected to be achieved in the next century.^2,3^ However, until this goal is achieved, there will still be many women with advanced cervical cancer who have limited treatment options.^4^ Exploring potential targeted therapies holds promise for the treatment of persistent, recurrent and metastatic cervical cancer.^4^

DIRAS family GTPase 1 (DIRAS1), also known as Rig (Ras-related inhibitor of cell growth), is a member of the small GTPase Ras superfamily.^5^ The *DIRAS1* gene, located on chromosome 19p13.3, consists of 2 exons and encodes a protein of 198 amino acids. Unlike common oncogenic small GTPases (e.g., Ras or Rho family members), DIRAS1 has been reported as a potential tumor suppressor in human renal cell carcinoma,^6^ ovarian cancer,^7^ colorectal cancer,^8^ gliomas,^9^ and esophageal squamous cell carcinoma.^10^ However, its expression pattern and role in cervical cancer remain unknown.

Epigenetic regulation mainly includes DNA methylation, histone modification and RNA modification, which are responsible for regulating gene expression and involved in normal biological functions and disease progression.^11^ Among them, DNA methylation is closely related to cancer progression, and mainly refers to the hypermethylation of CpG island in the promoter of key anti-oncogenes in order to interfere with the reading of DNA information, thus silencing the expression of key anti-oncogenes.^12^ Different histone marks determine the state of chromatin as well as the transcriptional activity of genes, both oncogenes and anti-oncogenes.^13^ N6-methyladenosine (m6A) RNA modification is the most abundant internal modification in eukaryotic mRNAs, regulated by methyltransferases (METTL3/METTL14) and demethylases (FTO and ALKBH5), and recognized by a set of “readers”, which decode the m6A and further regulate the degradation, stability, translation initiation, and translation efficiency of the modified mRNAs.^14^

In summary, this study will examine the anti-cancer function of DIRAS1 and its unique expression pattern in cervical cancer, and analyze the epigenetic regulatory mechanisms underlying the down-regulation of its expression.

## Materials and methods

### Cell lines and culture

Human cervical cancer cell lines (C33A and SiHa) were purchased from ATCC, and cultured in DMEM (KGM12800N–500, KeyGEN BioTECH) supplemented with 10% FBS (A31608, Gibco), 100 U/mL penicillin and 100 µg/mL streptomycin (KGY0023, KeyGEN BioTECH) in an incubator at 37°C with 5% CO_2_. 5-Azacytidine (5-aza-dC) (HY-10586)、Synonyms (SAHA) (HY-10221) and FB23–2 (HY-127103) were purchased from MCE.

### Plasmids and transfection

In loss-of-function experiments, the pLKO.1-puro empty vector (SHC001, Sigma-Aldrich) was used as a negative control (NC), and the shRNAs targeting sequences as shown in Table 1 were designed and synthesized by Sigma-Aldrich. In the gain-of-function experiments, pcDNA3.1 empty vector (VT1001, YouBio) was used as the NC, and human DIRAS1 (NM_145173) cDNA sequence was cloned into pcDNA3.1 vector to constitute a recombinant expression plasmid. All plasmids were transiently transfected into cells using Lipofectamine 2000 (11668019, Invitrogen) according to the manufacturer’s instructions.

**Table 1.** shRnas targeting sequences.

Names	Targeting sequences
shDIRAS1	CTACAAGCTCATCGTGCAGAT
shMETTL3	GCCTTAACATTGCCCACTGAT
shMETTL14	CCATGTACTTACAAGCCGATA
shALKBH5	GAAAGGCTGTTGGCATCAATA
shFTO	TCACGAATTGCCCGAACATTA

### Protein preparation and western blot

Cells were fully lysed with ice-cold strong RIPA lysate (CW2333S, CWBIO) on ice for 10 min. Cell lysates were centrifuged at 12,000 g for 10 min at 4°C to remove impurities. The supernatants after centrifugation were the protein solutions, and protein concentrations were quantified by BCA protein assay kit (CW0014S, CWBIO). Equal amounts of proteins from each group were electroblotted and separated by SDS-PAGE and electrotransferred onto PVDF membranes (0.45 μm, IPVH00010; 0.2 μm, ISEQ00010; Millipore). After blocking with 5% skimmed milk, the membranes were incubated overnight at 4°C with primary antibodies including DIRAS1 (ab65139, Abcam), METTL3 (ab195352), METTL14 (ab220030), ALKBH5 (ab195377), FTO (ab126605) and GAPDH (ab8245). Subsequently, the membranes were incubated with the corresponding secondary antibody at room temperature for 2 h. The immunoreactive bands were visualized by enhanced chemiluminescence (RPN2105, Amersham), and the gray values of the bands were read by Image J software.

### Cell counting kit-8 (CCK-8) assay

Cells were implanted into a 96-well plate at a density of 5 × 10^3^ cells/well 24 h after transfection, and cultured overnight. Cell viability was determined at 0 h, 24 h, 48 h, and 72 h of culture using CCK-8 (PF00004, PTG). The specific operations were as follows. Aspirate off the old culture medium and rinse the cells twice with PBS. 10% CCK-8 reagent (10 μL CCK-8 + 90 μL medium) was added back into the cell culture wells and incubation was continued for 2 h in the incubator. At the end of the incubation, the absorbance (OD) value of each well solution at 450 nm was measured using a microplate reader. The curve was plotted with time as the X axis and OD value as the Y axis.

### Colony formation assay

After 24 h of transfection, cells were seeded in a 6-well plate at 500 cells per well and cultured for 2 weeks. At the end of the culture, the cells were fixed with 75% ethanol for 30 min and stained with 0.2% crystal violet. Cell clones were then observed and counted.

### Transwell assay

5 × 10^5^ cells after 24 h of transfection were inoculated in the upper chamber of the Transwell. The Transwell was placed in a 24-well plate, and 100 µL of FBS-free medium was added to the upper chamber, and 600 µL of complete medium with 10% FBS was added to the lower chamber to induce cell migration or invasion. In addition, the Transwell membrane needs to be coated with Matrigel in the invasion assay. After 24 h of culture, cells migrating or invading under the membrane of the chamber were fixed with 4% paraformaldehyde for 15 min, and stained with crystal violet. Finally, the cells were counted and photographed under a microscope.

### RT-qCR

Cells were lysed using Trizol reagent, and total cellular RNA was extracted using RNA Extraction Kit (CW0581S, CWBIO). RNA was reverse transcribed to cDNA using the PrimeScript RT Master Mix (CW2569M, CWBIO). RT-PCR was performed with an SYBR Green master mix reagent (CW0957M, CWBIO). Β-actin was used as an internal reference, and relative gene expression was calculated using the 2^−ΔΔCT^ method. The primer sequences used were designed and synthesized by ORIGENE as follows: DIRAS1 F 5’-CCTTCATCCTGGTGTTCTCCGT-3’, R 5’-CTGCGTCTCATCGCACTTGTTG-3’; β-actin F 5’-CACCATTGGCAATGAGCGGTTC-3’, R 5’-AGGTCTTTGCGGATGTCCACGT-3’.

### Tissue microarray and immunohistochemical (IHC) analysis

Human cervical cancer tissue microarrays (ZL-Utr961) were obtained from Shanghai Biotechnology (Shanghai, China). Tissue sections were dewaxed and rehydrated by Van-Clear eco-friendly transparency (Shanghai Hongz Industrial Co., Ltd.) and gradient ethanol, then immersed in citrate buffer and microwaved for antigen repair. After sealing with 5% goat serum solution for 1 h at room temperature, the sections were incubated with anti-DIRAS1 solution (1:200; 12634–1-AP, Proteintech) overnight at 4°C, followed by incubation with goat anti-mouse/rabbit IgG polymer. Immunopositive staining on the sections was visualized with DAB chromogenic solution (DAB-2031, Fuzhou Maixin Biotechnology Development Co., Ltd.). Then, the sections were restained with hematoxylin solution and 1% hydrochloric acid solution. After dehydration with gradient ethanol and Van-Clear transparency, the sections were sealed with neutral gum and coverslips. The results were visualized with a microscopy and photographed (magnification 100× and 400×, respectively). All image processing was done using Image Pro Plus 6.0 software.

Results were scored at 400× field of view with 3–5 fields of view selected for each sample. Scoring was done independently by 3 experienced pathologists and the final results were combined by the 3 experimenters. The scale was based on staining intensity x staining area (0–9 points). The intensity of staining was categorized into 0–3 points: no staining (0 points), mild staining (1 point), moderate staining (2 points), and strong staining (3 points). The area occupied by positively stained cells was categorized into 0–3: unstained (0 points), <33% positively stained cells (1 point), 33–66% positively stained cells (2 points), >66% positively stained cells (3 points).

### Statistical analysis

The results of three independent replicate experiments were analyzed using SPSS 22.0 software, and presented as mean ± SD. Differences between the two groups were compared using the Student’s t-test, and a difference of *p* < .05 was considered statistically significant.

## Results

### Knockdown of DIRAS1 significantly promotes proliferation, growth and motility of C33A and SiHa cells in vitro

**Figure 1.**
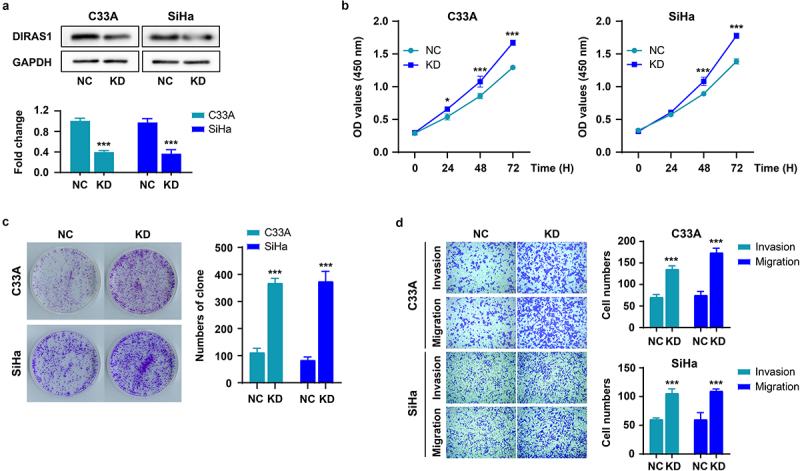
Knockdown of DIRAS1 significantly promotes proliferation, growth and motility of C33A and SiHa cells.

Firstly, DIRAS1 expression was significantly knocked down (KD) in C33A and SiHa cells cultured *in vitro* using targeted shRNA plasmid transfection (Figure 1a). Next, the effects of DIRAS1 knockdown on cell proliferation, growth, and motility were detected using CCK-8, clone formation and Transwell assays, respectively. As shown in Figure 1b, after 48–72 h of culture, the OD values at 450 nm of the KD group were significantly higher than that of the NC group, indicating that the cell number in the KD group was significantly greater than that in the NC group. In addition, the number and size of clones formed in the KD group were significantly higher than those in the NC group (Figure 1c). These data suggest that knockdown of DIRAS1 significantly promoted the proliferation and growth of C33A and SiHa cells *in vitro*. Moreover, the number of cells crossing the membrane (and Matrigel) and accomplishing migration or invasion was significantly greater in the KD group than in the NC group, indicating that DIRAS1 knockdown significantly promoted the motility of C33A and SiHa cells *in vitro* (Figure 1d).

The protein expression of DIRAS1 (a), the absorbance values at 450 nm of cells (b), the formed clones (c), the cells completed migration or invasion (d) in knockdown (KD) group of C33A and SiHa cells transfected with targeted shRNA plasmid. ***p < .001.

### DIRAS1 overexpression significantly inhibits the proliferation, growth and motility of C33A and SiHa cells in vitro

**Figure 2.**
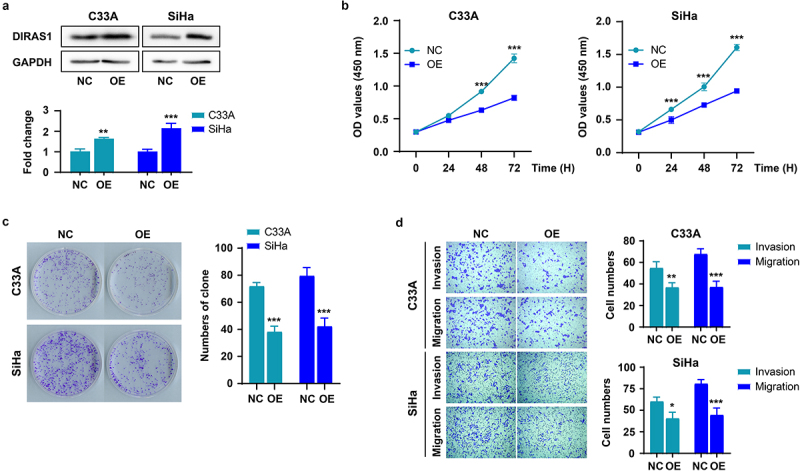
DIRAS1 overexpression significantly inhibits the proliferation, growth and motility of C33A and SiHa cells.

Along the lines of Figure 1, the expression of DIRAS1 was significantly up-regulated in C33A and SiHa cells using expression plasmid transfection (Figure 2a), and the effects of its overexpression (OE) on cell proliferation, growth, and motility were further examined. As shown in Figure 2b–d, overexpression of DIRAS1 significantly inhibited the proliferation, growth and motility of C33A and SiHa cells.

The protein expression of DIRAS1 (a), the absorbance values at 450 nm of cells (b), the formed clones (c), the cells completed migration or invasion (d) in overexpression (OV) group of C33A and SiHa cells transfected with pcDNA3.1 -expressing recombinant plasmid. *p < .05, **p < .01, ***p < .001.

### Altered expression pattern of DIRAS1 in human cervical cancer tissues

**Figure 3.**
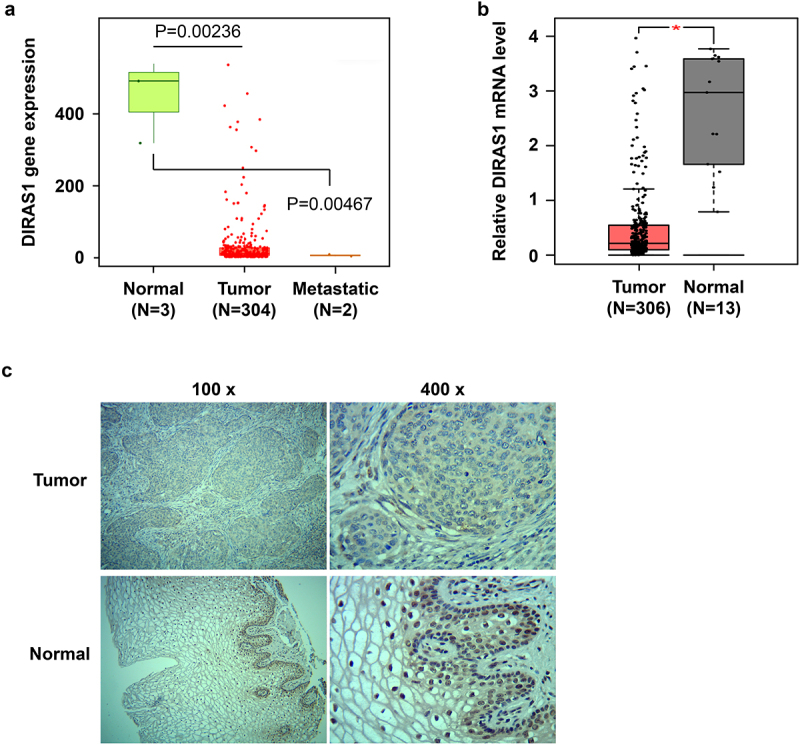
Altered expression pattern of DIRAS1 in cervical cancer tissues.

We analyzed RNA-Seq data of cervical squamous cell carcinoma and endocervical adenocarcinoma (CESC)-related tissues from the TNMplot database (tnmplot.com), and found that DIRAS1 mRNA levels were significantly reduced in CESC tissues (*N* = 304), compared to normal cervical tissues (Figure 3a). This was despite the fact that there were only 3 cases of normal cervical tissues and only 2 cases of CESC metastatic tissues (Figure 3a). Therefore, we analyzed DIRAS1 mRNA levels in CESC tissues again using the GEPIA database (gepia.cancer-pku.cn), which contains 306 cases of CESC tissues and 13 cases of normal cervical tissues from TCGA and GTEx. Consistent with the results of the data analysis of TNMplot, the results of data analysis from GEPIA also showed significantly lower DIRAS1 mRNA levels in CESC tissues (*N* = 306) compared to normal cervical tissues (*N* = 13) (Figure 3b).

(A). DIRAS1 mRNA levels in normal cervical tissues, cervical squamous cell carcinoma and endocervical adenocarcinoma (CESC) tissues, and CESC metastatic tissues. RNA-Seq data from the TNM database. (B) DIRAS1 mRNA levels in normal cervical tissues and CESC tissues. Data from the GEPIA database. (C) IHC analysis of DIRAS1 on cervical cancer tissues and para-cancerous tissues. *p < .05.

The results of IHC analysis performed on tumor tissues and para-cancerous tissues from 48 clinically collected patients with cervical cancer showed that, consistent with the mRNA levels, DIRAS1 protein expression was also significantly reduced in cervical cancer tissues compared to para-carcinoma tissues (Table 2 and Figure 3c). In addition, in para-carcinoma tissues, DIRAS1 protein showed significant nucleus accumulation (Figure 3c). In contrast, in cervical cancer tissues, DIRAS1 protein expression was not only significantly reduced, but the nucleus accumulation also disappeared significantly (Figure 3c). Furthermore, the expression level of DIRAS1 in tumor tissues was significantly negatively correlated with the pathological grade of cervical cancer patients (Table 3).

**Table 2.** DIRAS1 expression in cervical cancer tissues compared with para-carcinoma tissues.

Group	n	DIRAS1 expression		
		Low (n%)	High (n%)	P
cervical cancer	48	32 (66.7)	16 (33.3)	0.004**
para-carcinoma	48	17 (35.4)	31 (64.6)

**Table 3.** DIRAS1 expression associated with the clinicopathological parameters in CCA.

clinicopathological parameters	n	DIRAS1 Low (n%)	DIRAS1 High (n%)	P
Age (years)				
≤55	23	17 (73.9)	6 (26.1)	.529
>55	23	14 (60.9)	9 (39.1)	
Tumor diameter (cm) ≤2 >2	18 28	13 (72.2) 18 (64.3)	5 (27.8) 10 (35.7)	.812
Pathological grading II III	23 23	11 (47.8) 20 (87.0)	12 (52.2) 3 (13.0)	.012*

### M6A modification promotes DIRAS1 translation

**Figure 4.**
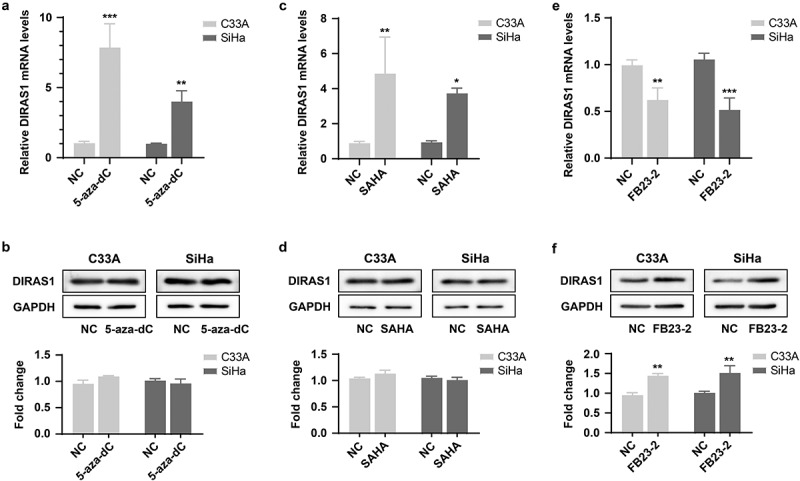
The mechanisms of epigenetic regulation to regulate DIRAS1.

Given the anti-oncogenic role of DIRAS1 and its specific expression downregulation in cervical cancer cells, we further explored the major epigenetic mechanisms that regulate the downregulation of DIRAS1 expression in cancer cells. The specific DNA methylation inhibitor 5-aza-dC (2 µM for 48 h) as well as the histone deacetylase inhibitor SAHA (1 µM for 48 h) were used to treat the cells. Their applications both resulted in a significant increase in DIRAS1 mRNA levels in C33A and SiHa cells (Figure 4a, c). Based on this result, we hypothesized that both DNA methylation and histone acetylation might be involved in the transcriptional regulation of DIRAS1 in cancer cells, with DNA methylation hindering DIRAS1 transcription and histone acetylation promoting DIRAS1 transcription. However, the results of western blot showed that the application of 5-aza-dC and SAHA did not significantly affect the intracellular DIRAS1 protein level (Figure 4b, d). This result suggests that there may be more critical post-transcriptional regulatory mechanisms to significantly reduce DIRAS1 protein levels in cervical cancer cells.

The specific DNA methylation inhibitor 5-aza-dC (a and b), histone deacetylase inhibitor SAHA (c and d) and FTO inhibitor FB23-2 (e and f) treated the, and the mRNA and protein levels of DIRAS1 in cells. *p < .05, **p < .01, ***p < .001.

**Figure 5.**
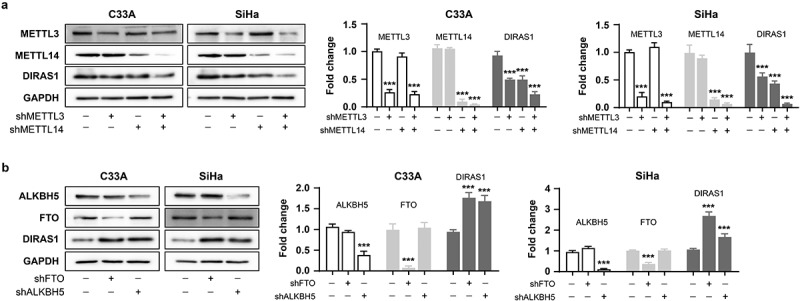
M6A modification promotes DIRAS1 translation.

Subsequently, the treatment of cells with the FTO inhibitor FB23–2 (1 µM for 48 h) resulted in significant down-regulation of intracellular DIRAS1 mRNA levels, but significant up-regulation of DIRAS1 protein levels (Figure 4e, f). This result implies that m6A modification may be the key post-transcriptional regulatory mechanism to regulate DIRAS1 mRNA degradation and translation. Furthermore, the expression of METTL3, METTL14, METTL3/METTL14, FTO and ALKBH5 were significantly downregulated using specific shRNA plasmid transfection, respectively (Figure 5). Downregulation of METTL3 and METTL14 expression significantly suppressed DIRAS1 protein expression, and the simultaneous downregulation of METTL3 and METTL14 expression had a synergistic effect (Figure 5a). Downregulation of FTO and ALKBH5 expression significantly increased DIRAS1 protein expression (Figure 5b).

The DIRAS1 protein levels in C33A and SiHa cells in which the expression of METTL3, METTL14, METTL3/METTL14 (a), FTO and ALKBH5 (b) were significantly downregulated using specific shRNA plasmid transfection, respectively. ***p < .001.

## Discussion

In this study, we investigated the role of DIRAS1 expression in the proliferation, growth and motility of cervical cancer cells cultured *in vitro* using knockdown and exogenous overexpression of DIRAS1 in C33A and SiHa cells. The results of Figures 1 and 2 indicate that DIRAS1 has a significant inhibitory effect on the proliferation, growth, and motility of cervical cancer cells cultured *in vitro*, which is consistent with its role in other types of solid tumors. Unfortunately, this study was not in a position to validate its role on the *in vivo* experiments. The results of this study, in combination with those of other studies, suggest that DIRAS1 has a potent cancer inhibitory effect. Focusing on DIRAS1 may be beneficial for the advancement of cancer treatment.

Regarding the mechanism of cancer inhibition of DIRAS1, DIRAS1 appears to inhibit cancer by competitively binding SmgGDS with other proteins with strong affinity.^15^ SmgGDS is a key promoter of tumorigenesis and cancer cell proliferation with cytoplasmic-nuclear shuttling. In the cytoplasm, SmgGDS binds to small GTPases and facilitates their transport to the plasma membrane.^15^ These small GTPases normally exert pro-oncogenic activity. In the nucleus, SmgGDS can regulate the expression of more than 600 gene products, including mainly proteins controlling the cell cycle, by interacting with transcription factor complexes.^16^ DIRAS1 is expressed in normal cells and binds to SmgGDS, blocking the action of SmgGDS with small GTPases or transcription factor complexes.^15,16^ In cancer cells, deletion of DIRAS1 expression eliminates this brake and allows SmgGDS to exert oncogenic activity.^17^ However, other mechanisms of cancer inhibition by DIRAS1 need to be further explored.

Furthermore, using RNA-Seq data as well as IHC, this study determined that DIRAS1 expression was significantly downregulated in cervical cancer tissues. The same trend was also found in other types of solid tumor tissues. Similar downregulation of DIRAS1 expression was found in renal cell carcinoma,^6^ ovarian cancer,^7^ colorectal cancer,^8^ glioma^9^ and esophageal squamous cell carcinoma^10^ tissues. The low-level expression of the DIRAS1 protein in tumor tissues may be the main reason for its lack of nuclear localization. Generally, the protein first accumulates in the cytoplasm before being translocated to the nucleus. For example, nuclear localization is also found to occur with high expression of DIRAS1 in IHC analysis of ovarian cancer tissues.^7^ The mechanisms mediating the nucleoplasmic shuttling of DIRAS1 protein remain to be explored. In addition, statistical analysis of the data showed that low expression of DIRAS1 was associated with high pathological stage in cervical cancer patients. A study indicates that low level of DIRAS1 is significantly associated with advanced clinical stage and lymph node metastasis in patients with esophageal squamous cell carcinoma.^10^ Unfortunately, this study failed to better complete patient follow-up and tracking to analyze the correlation between DIRAS1 expression and patient prognosis. However, one study has reported that downregulation of DIRAS1 expression is associated with decreased disease-free survival and overall survival in ovarian cancer patients.^7^ In addition, it has been suggested that DIRAS1 is a good predictor of overall survival in HER2+ breast cancer patients receiving neoadjuvant chemotherapy.^18^

The focus of this study is on the regulatory mechanisms underlying the downregulation of DIRAS1 expression in cancer cells. Current mutation analysis did not identify inactivating mutations on the *DIRAS1* coding region.^9^ Heterozygous deletion of the *DIRAS1* gene is present in esophageal squamous cell carcinoma tissues.^10^ The present study was not equipped to detect mutations in *DIRAS1* occurring in cervical cancer tissues and mainly focused on epigenetic regulation. Most current studies have also focused on the epigenetic regulation mechanisms of DIRAS1 expression, mainly DNA methylation and histone modification. Aberrant hypermethylation of CpG islands in the promoter region of *DIRAS1* is identified by bisulfite sequencing in renal cell carcinoma cell lines (ACHN, 786-O and Caki-1).^6^ Hypermethylation of the *DIRAS1* promoter region and the resulting deletion or reduction of DIRAS1 expression have also been identified in colorectal cancer samples, esophageal squamous cell carcinoma tissues, and corresponding cell lines.^8,10^

In addition, experimental data have shown that 5-aza-dC treatment of renal cell carcinoma cell lines (ACHN, 786-O, and Caki-1) (10 µM for 96 h),^6^ colorectal cancer cell lines (2 µM for 96 h, with fluid changes every 24 h),^8^ glioblastoma cell lines (U251MG and Hs683) (1 µM for 72 h)^9^ and esophageal squamous cell carcinoma cell lines (KYSE30 and KYSE510)^10^ result in a significant elevation of DIRAS1 mRNA levels. Treatment of both glioblastoma cell lines^9^ and renal cell carcinoma cell line (UOK146)^19^ with histone deacetylase inhibitors also result in a significant increase in DIRAS1 mRNA levels. These findings seem to suggest that DIRAS1 expression is regulated by DNA methylation and histone modifications. However, none of these studies detected changes in DIRAS1 protein levels after inhibitor treatment.

In the present study, we also treated C33A and SiHa cells with 5-aza-dC and histone deacetylase inhibitors and found that DIRAS1 mRNA levels were significantly increased after inhibitor treatment. However, western blot results showed that DIRAS1 protein levels were not significantly altered after inhibitor treatment, suggesting the existence of a more critical post-transcriptional regulatory mechanism for DIRAS1 that further controls the low-level expression of DIRAS1 protein in cancer cells. The application of the FTO inhibitor FB23–2 as well as the knockdown of FTO and ALKBH5 significantly reduced intracellular protein expression of DIRAS1, while knockdown of METTL3/14 significantly increased intracellular DIRAS1 protein expression. This result suggests that the m6A modification mechanism may be involved in the low-level expression of DIRAS1 protein in cancer cells. To the best of our knowledge, studies related to DIRAS1 mRNA m6A modification are lacking. However, the results of this study are still preliminary and fail to clarify the specific m6A modification sites of DIRAS1 mRNA and how m6A modification affects the altered mRNA and protein levels of DIRAS1.

One study evaluates the total mRNA m6A levels in 286 pairs of cervical cancer samples and their adjacent normal tissues, and find that the total mRNA m6A levels in cervical cancer tissues are significantly lower compared with adjacent normal tissues, m6A level reduction is also significantly correlated with FIGO stage, tumor size, degree of differentiation, lymphatic invasion, cancer recurrence and patient survival.^20^ In addition, some studies have reported that the expression of FTO and ALKBH5 is significantly up-regulated in cervical cancer and significantly enhances the malignant phenotype of cervical cancer cells.^21–23^ The pro-cancer mechanism of FTO and ALKBH5 may also include the removal of m6A modification of DIRAS1 mRNA, which reduces the DIRAS1 protein levels.

What cannot be ignored is the recognition and interpretation of RNA m6A modifications by m6A “readers”, which really affects the fate of RNA. An m6A “reader” recognizes and binds an m6A site and may mediate different biological processes and changes. For example, YTHDC1 affects pre-mRNA splicing^24^ and nuclear export.^25^ YTHDC2 accelerates the translation and decay of mRNAs.^26^ YTHDF1 enhances the translation efficiency of mRNAs, whereas YTHDF2 promotes the degradation of mRNAs.^27,28^ YTHDF3 promotes the production of proteins through its interactions with YTHDF1, and influences mRNA decay through YTHDF2.^29^ IGF2BP1/2/3 improves mRNA stability and translation efficiency.^30^ Combined with the result that treatment with FTO inhibitors decreased DIRAS1 mRNA levels but increased DIRAS1 protein expression, we hypothesized that m6A modification of DIRAS1 mRNA at least mediated the degradation or decay of its mRNAs while promoting its translation. However, as mentioned earlier, this study was unable to identify the specific m6A modification sites of DIRAS1 mRNA, as well as the specific “readers” that recognize the m6A and the specific roles they mediate.

In summary, DIRAS1 exerts a significant anti-oncogenic function and its expression is significantly downregulated in cervical cancer cells. m6A modification is a key mechanism to regulate DIRAS1 translation and protein levels.

## Data Availability

The datasets generated during and/or analyzed during the current study are not publicly available, but are available from the corresponding author on reasonable request.
